# Simultaneous and Rapid Detection of *Salmonella typhi*, *Bacillus anthracis*, and *Yersinia pestis* by Using Multiplex Polymerase Chain Reaction (PCR)

**DOI:** 10.5812/ircmj.9208

**Published:** 2013-11-05

**Authors:** Nargess Safari Foroshani, Ali Karami, Fatemeh Pourali

**Affiliations:** 1 Science and Research Islamic Azad University, Tehran, IR Iran; 2 Research Center of Molecular Biology, Baqiyatallah University of Medical Sciences, Tehran, IR Iran

**Keywords:** Salmonella typhi, Bacillus anthracis, Yersinia pestis, Multiplex PCR

## Abstract

**Background:**

*Salmonella typhi*, *Bacillus anthracis*, and *Yersinia pestis* are some serious human pathogens, which their early diagnosis is of great importance. *Salmonella typhi*, *Bacillus anthracis*, and *Yersinia pestis *cause typhoid fever, anthrax, and plague respectively. These bacteria can be used to make biologic weapons.

**Objectives:**

In this study, we designed a new and rapid diagnostic method based on Uniplex and Multiplex PCR method.

**Materials and Methods:**

Uniplex and multiplex Polymerase Chain Reaction (PCR) were conducted on virulent genes of hp and invA of *Salmonella typhi*murium, Pa and chr of *Bacillus anthracis*, and pla of Yersinia pestis. A genome from other bacteria was used to study the specificity of the primer and the PCR test.

**Results:**

Standard strains used in this study showed that primers were specific. As for sensitivity, it was shown that this method can diagnose 1-10 copies of the genome, or 1-10 Colony Forming Units (CFU) for each of the bacteria. All pieces except anthrax were sequenced in PCR to validate the product. DNA fragment resulted from *Bacillus anthracis* was confirmed by restriction enzyme digestions.

**Conclusion:**

The designed methods are accurate, rapid, and inexpensive to find and differentiate these bacteria from similar bacteria. They can be applied for rapid diagnosis of these agents in different specimens, and bioterrorism cases.

## 1. Background

*Salmonella typhi*, *Bacillus anthracis*, and *Yersinia pestis* are known as group A pathogens, which have a high potential for human pathogenicity. To deal with these pathogens, rapid detection and treatment are very important. *Salmonella typhi* causes typhoid, with a morbidity of more than 16 million people worldwide, and a mortality of about 600 thousand deaths (1). All salmonellas are pathogenic for humans, mammals and birds except *Salmonella typhi*, which causes diseases in humans and higher primates only. Therefore, it is transmitted through water, food, feces and urine of infected humans, mammals and birds (2). Anthrax is a zoonotic infectious disease, caused by *Bacillus anthracis*. This disease is found worldwide, and its bacteria can be transmitted to humans indirectly from contaminated livestock products or directly by the spores of *Bacillus anthracis*. Four forms of anthrax include cutaneous, pulmonary, gastrointestinal, and meningitis. Due to its special features, sporogenesis, high virulence, and ease of mass production and application, this bacterium tops the biological and bioterrorism weapons (3). Plague is a dangerous and deadly disease caused by bacterium *Yersinia pestis *in most rodents and humans (4). The gram-negative coco-*Bacillus* does not have spores and movement, which is normally transmitted in enzootic form among rodents such as mice (5). The disease agent can be transmitted to humans through infected ectoparasites bite, particularly fleas (6, 7). Three clinical manifestations of disease include bubonic, septicemic, and pneumonic. Its pneumonic type causes the most severe clinical manifestation, entails high mortality, and is easily transmitted from person to person (8). Blood culture, bone marrow, rectal swabs, stool cultures, urine cultures, and other tests such as ELISA and immune-florescent are used for laboratory diagnosis (9, 10). Identifying the pathogenic agent of anthrax is difficult and time consuming due to its similarity to nonpathogenic bacilli in soil, and just advanced and reference laboratories can identify it accurately. Meanwhile, in the conventional methods, it is essential to determine the virulence in laboratory animals, which requires special biosafety facilities and equipment for dealing with hazardous agents, in addition to be time consuming, and also exposes health workers in this field at risk (11). Biochemical tests, susceptibility to specific bacteriophages, inoculation to laboratory animals, and culture in specific media are used for the diagnosis of plague agent. Furthermore, samples can be dried and contaminated or its bacteria can die due to the lack of appropriate means of transferring samples from infected areas to diagnostic centers (12). All the usual bacteriological methods like culture and biochemical tests as gold standard for identification of infectious disease agents are time-consuming and useless for rapid and accurate diagnosis of the disease (13). Meanwhile in molecular methods, the virulent infectious agent can directly and rapidly be diagnosed without the need for such investigations. Thus, conventional methods are worthless in biological and bioterrorism attacks, and we need rapid diagnostic methods which can help apply therapeutic and preventive measures in few hours. These methods are also widely used in clinical laboratories. Inability to quickly identify these biological agents means that they are life-threatening as nuclear weapons, and even more dangerous, because they can be transmitted to other areas and even the whole world (14).

## 2. Objectives

In this study, we designed a new and rapid diagnostic method based on Uniplex and Multiplex PCR method.

## 3. Materials and Methods

The standard strains were purchased from the Reference Laboratory of the Ministry of Health and Pasteur Institute in Tehran. Due to hazards of working with virulent strains, nonvirulent *Bacillus anthracis *strains, known as Stern or livestock vaccine strain was used, which has no difference with the wild strain regarding microbiologic features. The original strains of *Staphylococcus aureus *, *Shigella sonnei *, *Escherichia coli *, *Enterococcus faecalis, Pseudomonas aeruginosa *, *Citrobacter freundii *, *Serratia marcescens, *and *Klebsiella pneumoniae *were used as negative control agents, which were purchased from laboratory of Pasteur Institute in Tehran. Used strains are shown in Table 1. Chemical materials were purchased from Merck Co., MgCl _2 _, buffer, nucleotides, and Taq polymerase enzyme were purchased from Cinnagen Co. In this research, Mastercycler gradient (Eppendorf Co., Germany) was used for thermal cycling. Small horizontal electrophoresis (Paya Pajouhesh Co., Mashhad, IR Iran), and its power supply with the buffer TBE 0.5 were used for electrophoresis. Gel was studied by UVIdoc (UVItec UK Co.). Standard molecular marker of 100 bp was purchased from Fermentas Co. 

**Table 1.** List of Control Agent Bacteria Used in This Study for Specificity Analysis

	Strain No.	Microorganism No.	Product Place
***Staphylococcus aureus***	ATCC	25923	IPR^a^
***Shigella sonnei***	ATCC	9290	IPR^a^
***Escherichia coli***	ATCC	25922	IPR^a^
***Enterococcus faecalis***	ATCC	29212	IPR^a^
***Pseudomonas aeruginosa***	ATCC	27853	IPR^a^
***Citrobacter freundii***	PTCC	1600	IPR^a^
***Serratia marcescens***	PTCC	1111	IPR^a^
***Klebsiella pneumonia***	ATCC	7881	IPR^a^

^a^ Pasteur Institute of Iran.

### 3.1. Primer Designing

The primers were designed for *Salmonella typhi *, based on two principle segments of *Salmonella typhi *genome, invA and hp. The primers for *Bacillus anthracis *were designed based on two principle segments of organism genome, pA and chr. The primers for *Yersinia pestis *were designed based on one principle segment of plague bacterium genome, pla. All primers were designed and made by Cinnagen Company. DNASIS software (HITACHI, TOKYO, Japan) Blast version 6.71, and OLIGO were applied to investigate sequence analysis and primer design. Also, the loci of primers on the genome, the size of the amplified fragment, and the target genes are shown in Table 2. The sequence of the primers used in this study is shown in Table 3. The primers were diluted according to manufacturer’s guidelines at concentration of 100 pm using injection distilled water, and then diluted to a concentration of 20 pm and maintained at -20°C. 

**Table 2.** Used Primers Profile

	Protein	Product Size, bp	Gene Statute, Plasmid/ chromosome	Primer No.
***Salmonella typhi***	invA	373	chromosome	S12-S13
***Salmonella typhi***	Hp	489	chromosome	-
***Bacillus anthracis***	pA	1083	Plasmid (PXO1)	125/126
***Bacillus anthracis***	Chr	164	chromosome	12-130
***Yersinia pestis***	Pla	520	Plasmid (pST1)	-

**Table 3.** Used Primers Sequence

	Primer No	Sequence of Primers
**-**	S12F	gta ttg ttg att aat gag atc cg
**-**	S13R	ata tta cgc acg gaa aca cgt t
***Salmonella typhi***	TF	tgt ccg ctg tct gaa gtc at
***Salmonella typhi***	TR	atc tca ggc aaa ctc aca agg g
***Bacillus anthracis***	125	tta atg cga ttg tct acg at
***Bacillus anthracis***	126	gat caa ttg cga ccg tac ttg aa
***Bacillus anthracis***	129	ccc agg ggg aca aac gat agc tcc
***Bacillus anthracis***	130	aac gat agc tcc tac att tgg ag
***Yersinia pestis***	Yer F	tgg act tgc agg cca gta tcg
***Yersinia pestis***	Yer R	cca tgc ctg aaa gac gtg gag

### 3.2. Cultivation and Extraction of DNA

Master bacteria were inoculated on solid culture and Luria liquid media, and cultivated at 37°C for 18-24 hours. In addition to studying the shape and structure of the colonies and especially the colony characteristics, gram-staining, and biochemical tests were conducted. Then, DNA genome was extracted by conventional method from 1.5 to 5 mL of liquid culture (15).

### 3.3. Performing Multiplex PCR

PCR was conducted to assess target genes of invA and hp for *Salmonella typhi *, Pa and chr for *Bacillus anthracis, *and pla for *Yersinia pestis *in standard reaction volume of 25 µL. One µL of genomic sample preparation was added to the tube containing the compounds listed in Table 4, and placed in Master Cycler (Eppendorf) using the following program: The first step included a cycle of 7 min at 94°C, and then 30 cycles of three steps, 94°C for 1 min, 55°C junction primers for 1 min, 72°C for 1 min, and a final step at 72°C for 7 min. Before performing multiplex PCR, each of the materials and procedures of PCR were optimized. After obtaining the most appropriate amount of material and the optimum conditions for multiplex PCR, the amounts listed in Table 4 were obtained. After completing amplification reaction, 0.5 X TBE buffer was run on electrophoresis with a voltage of 100 V. Finally, to see the band, agarose gel was stained with ethidium bromide, and after rinsing, it was studied in the UV system. Reactions for all three bacteria were performed both in uniplex and multiplex ways. 

**Table 4.** Quantities and Amounts of Compounds Used in Multiplex PCR Process

	Materials	Amount, µL
**1**	Distilled Water	17.6
**2**	Buffer 10X	2.5
**3**	MgCL_2_, 50 mM	1
**4**	dNTPmix, 100mM each	0.5
**5**	Primer F	1
**6**	Primer R	1
**7**	DNA	1
**8**	Taq	0.4
**Total**		25

### 3.4. Determining Specificity

PCR was performed under the same conditions on the extracted genome of negative control bacteria to obtain the specificity of gene primers invA and hp of *Salmonella typhi*, pA and chr of *Bacillus anthracis*, and pla of *Yersinia pestis*, and also detecting the orientation of the bacteria. 

### 3.5. Determining Sensitivity

Isolated colonies of bacteria causing typhoid and plague, and nonvirulent strain of *Bacillus anthracis* were inoculated in a flask containing 50 mL of broth, and placed in shaker incubator for 24 hours at 37°C. The next day, 10 tubes were prepared containing 900 µL of sterile broth, and 100 µL of each bacterial culture was added to the first tube (dilution of 10^-1^), and this dilution continued to the tube number 10. Simultaneous with preparing dilutions, 200 µL of each dilution was added to solid medium plates, and it was evenly spread on the media. The plates were kept at 37°C during the night. After complete growth, plates with 30 to 300 colonies were counted and measured. The experiment was repeated 3 times to increase the accuracy. Simultaneous with preparing dilutions, 2 µL of each tube was added to PCR tubes containing necessary compounds, and was tested at binding temperature of 55°C.

### 3.6. Studying Sensitivity With Concentration of Genome

The concentration of extracted genome was measured at 260 and 280 nm using spectrophotometer. Then, it was performed at various concentrations of PCR genome. 

### 3.7. Verification of PCR Product

This was performed by restriction digestion of PCR product or its sequencing. For *Bacillus anthracis* PCR products we used HindIII digestion. Five uL of PCR product was added to the tube containing 1 µL of HindIII enzyme and enzyme buffer. Sterile distilled water was added to reach the final volume of 20 µL, and this mixture was maintained for 2 hours at 37°C. Then, 10 µL of this compound was investigated on agarose gel, and the other amplified products along with their front and rear primers were sent to the relevant company for sequencing.

### 3.8. Bioinformatic Analysis of Sequence

Incoming records containing sequences of primers F and R for each segment were investigated regarding accuracy and overlap with each other. Due to the small size of the parts, it was expected to have much overlap. DNASIS Ver 2.6 (Hitachi Co.) was used to identify overlapping. To this end, text file of primer F was compared with reverse complement sequences of R primer. By using the program ALIGN, they were attached together and edited to make the original sequence for each segment. Obtained sequences were compared with gene banks to find similarities and differences by using BLAST 2.2.9 (16). The DNASIS software was used to compare two sequences resulted from this study with reference sequences to identify the similarities and differences between the nucleotides.

## 4. Results

### 4.1. Uniplex and Multiplex PCR of Standard Strains

**Figure 1.**
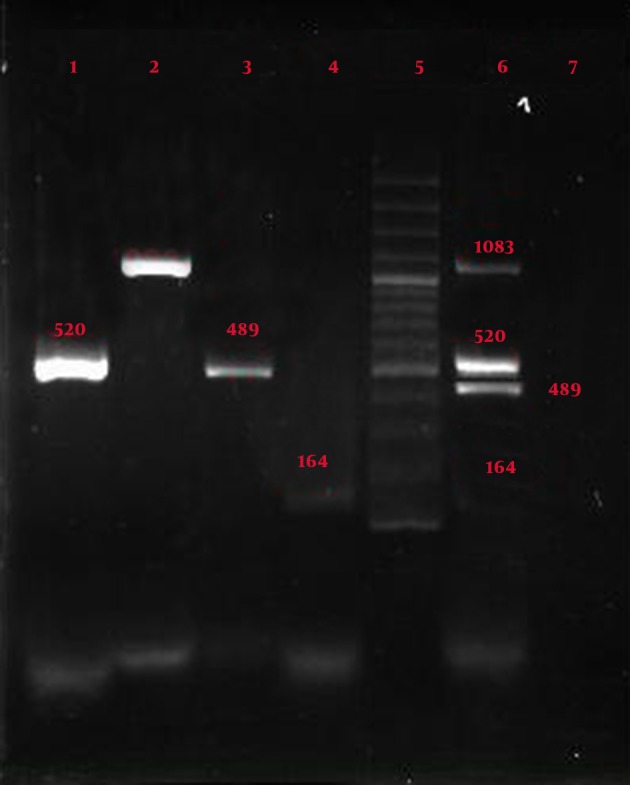
Result of Uniplex and Multiplex PCR Lane1: *Yersinia pestis* by primer Yer (size 520 bp); Lane2: *Bacillus anthracis* by primer 125 (size 1083 bp); Lane3: *Salmonella typhi* by primer T (size 489 bp); Lane 4: *Bacillus anthracis* by primer 129 (size 164 bp); Lane 5: Molecular marker (100, 200, …..500, …1000, 1100, 1200, 1300, 1400); Lane 6: Multiplex PCR with the 4 pairs special primers, *Yersinia pestis* by primer Yer*, Bacillus anthracis *by primer 125, *salmonella pestis* by primer T, *bacillus anthracis* by primer 129; Lane 7: Negative control

**Figure 2.**
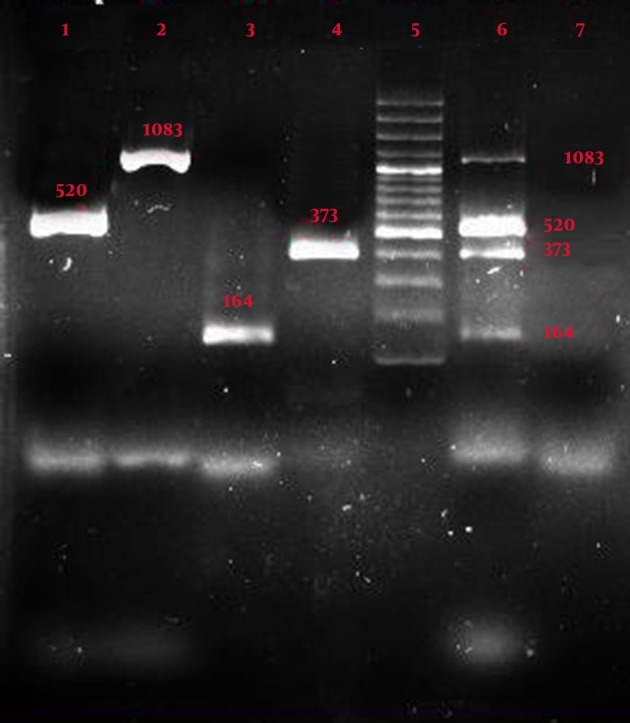
Result of Uniplex and Multiplex PCR with the 4 Pairs Special Primers Lane 1: *Yersinia pestis* by the primer Yer (size 520 bp); Lane 2: *Bacillus anthracis* by the primer 125 (size 1083 bp); Lane 3: *Bacillus anthracis* by the primer 129 (size 164 bp); Lane 4: *Salmonella typhi* by the primer S12; Lane 5: Molecular marker; Lane 6: Multiplex PCR by the 4 pairs special primers: *Bacillus anthracis*( primer 129), *Salmonella typhi*(primer S12), *Yersinia pestis *(primer Yer), *Bacillus anthracis *(primer 125); Lane 7: Negative control

As seen in Figure 1, result of comparing the samples with four pairs of primers showed that the primers created 4 bands in uniplex and multiplex forms with sizes 164, 489, 520, and 1083 bp with primers 125 and 129 of *Bacillus anthracis *, primer T of *Salmonella typhi, *and primer F of *Yersinia pestis *. Figure 2 shows samples with four pairs of primers, which created 4 bands in uniplex and multiplex forms with sizes 164, 373, 520, and 1083 bp with primers 125 and 129 of *Bacillus anthracis *, primer S12 of *Salmonella typhi, *and primer F of *Yersinia pestis *. 

**Figure 3.**
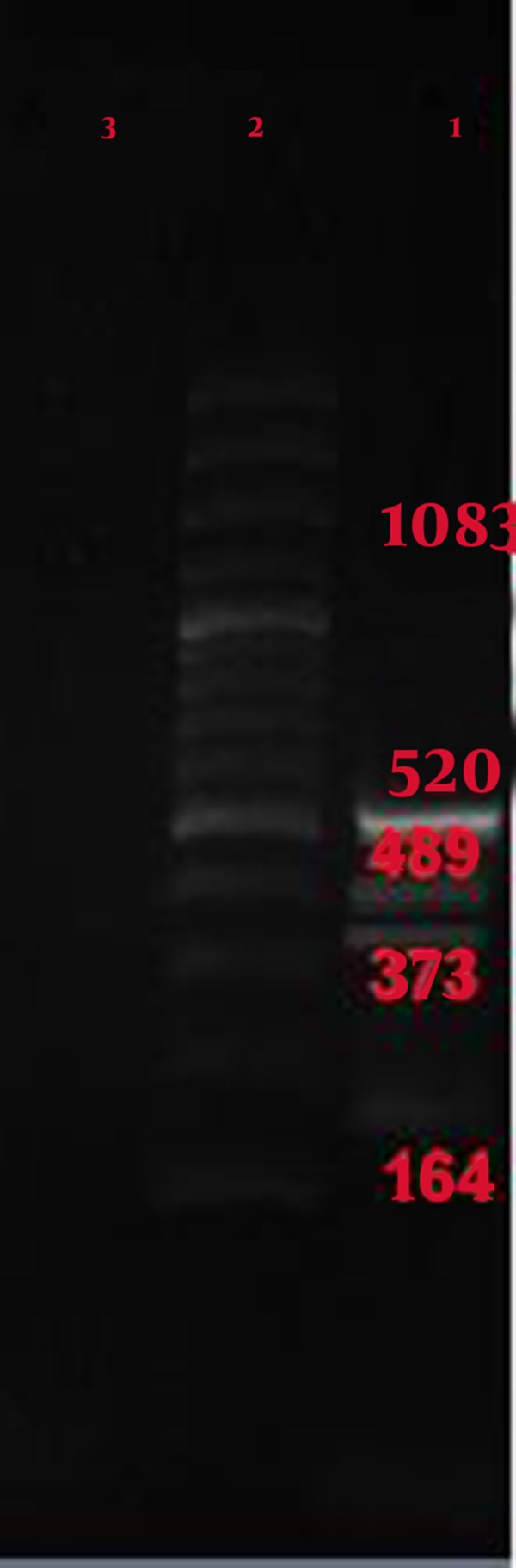
Result of Multiplex PCR with 5 Pairs Specials Primer Lane 1: multiplex PCR, *Bacillus anthracis* with primer 129 (size 164 bp), *Salmonella typhi* with primer S12 (size 373 bp), *Salmonella typhi* with primer T (size 489 bp), *Yersinia pestis* with primer Yer (size 520 bp), and *Bacillus anthracis* with primer 125 (size 1083 bp); Lane 2: ladder; Lane 3: Negative control

Figure 3 shows multiplex PCR with all three standard strains of bacteria, conducted with 5 pairs of specific primer PCR, and shows the presence of all expected components. 

### 4.2. The Specificity of PCR

Figures 1, 2, and 3 show the specificity of multiplex PCR and primers in that specific primers of Yersinia pestis, *Bacillus anthracis *, and *Salmonella typhi *did not create any bands with *Staphylococcus aureus *, *Shigella sonnei *, *Escherichia coli *, *Enterococcus faecalis*, *Pseudomonas aeruginosa *, *Citrobacter freundii *, *Serratia marcescens, *and *Klebsiella pneumoniae *. 

### 4.3. Confirming the R Product of Bacillus anthracis Multiplex PCR With Primer 125

**Figure 4.**
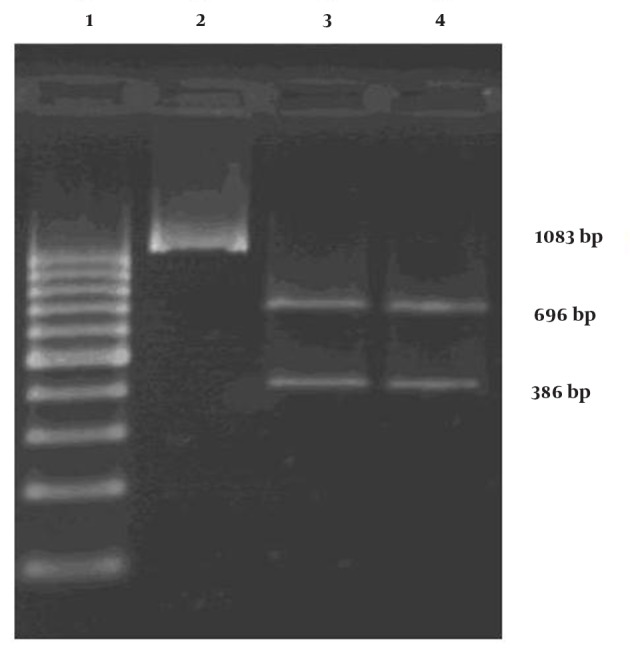
HindIII Restriction Digestion of PCR Product Lane1: Molecular marker; Lane 2: PCR fragment; Lane 3: Product digested by HindIII enzyme; Lane4: Product digested by HindIII enzyme

Results from enzyme cleavage product 1083 bp by the restriction enzyme cutting effect of HindIII, create 2 products with sizes of 696bp and 386bp, which confirm the accuracy of product generated by the primers (Figure 4). 

## 5. Discussion

Conventional methods to detect *Salmonella typhi* is a time consuming process without enough efficiency for rapid detection of bacteria (9). PCR has been used for detection and differentiation of typhoid from other enterobacteriaceae, as well as other salmonellas (17). To identify *Salmonella typhi* molecularly, researchers have designed and investigated several primers based on known gene sequence of tyv, flic-d, flic-a, viaB, prt, and spvC (18). In a study, researchers were able to detect *Salmonella typhi* on five pairs of primers based on genes viaB, fliC, tyv (rfbE), and prt (rfbS), by using multiplex method (19). The number of primers for this method is high, and yet the methods used is normal PCR at conventional speed. A variety of methods have been used for the detection of *Bacillus anthracis* in clinical animal and environmental specimens. Standard conventional detection methods are time consuming and inefficient for rapid detection of bacteria (20-22). 

Different molecular methods have been used to detect *Bacillus anthracis,* and differentiating it from other bacilli (23-28). Hinbaj and Shwamm (1993) used gene primers pla to identify certain bacteria in their studies of bacterial genomes for *Yersinia pestis* under different species from Asia, Africa and America. Their research showed that performing PCR is reliable for control and diagnostic purposes, and epidemiological studies (29). Angeltaler et al. compared PCR method with inoculation to laboratory animals method to identify the bacteria causative for plague. Their research showed that not only PCR method eliminates false negative problem due to resistance of some laboratory animals against *Yersinia pestis*, but also is cost and time effective. Therefore, they concluded that this method is more sensitive and accurate compared to conventional diagnostic methods such as bacteriology cultivate and inoculation to laboratory animals, and can be a suitable method for detection, control and surveillance of plague (30). Multiplex method is not only less expensive and faster than uniplex, but also removes false negatives completely. Janse et al. performed multiplex real time PCR to detect *Bacillus anthracis*, *Francisella tularensis,* and *Yersinia pestis*. They used *B. thuringiensis* spores as internal controls. In another study by Skottman et al. (31) multiplex real time PCR was performed for simultaneous detection of *Bacillus anthracis*, *Francisella tularensis* and *Yersinia pestis *. Also, they declared that simple multiplex PCR is much easier than real time PCR. In other study conducted by Skottman et al. (32) they applied multiplex PCR and RT-PCR enzyme hybridization assays for simultaneous detection of CDC category “A” bioterrorism agents is a complex and difficult method for rapid detection, and needs more simplification for filed application.

In this research performed based on invA and hp genes of *Salmonella typhi*, pA and chr of *Bacillus anthracis*, and pla of *Yersinia pestis*, all these genes were identified according to their specific primers. Due to the enormous progress in the field of molecular methods, such as multiplex PCR, they can be used to detect a variety of infectious agents, including detection of these three, i.e. *Bacillus anthracis*, *Salmonella typhi*, *Yersinia pestis*. The major factor in practical application of PCR is identification of specific primers. Primer designing needs studying different loci so that researchers used different existing parts of the sequences to design appropriate primers and probes. In this study, standard samples were used. Selected primers were able to identify the genomes of the virulent agents of these three bacteria specifically, and in direct samples. They neither respond to similar bacteria, nor to other bacteria that might exist in clinical samples. The size of PCR product of 164, 373, 489, 520, 1083 bp can be easily detected with usual agarose gel electrophoresis at concentration of 1% to 1.5%.

This approach allows rapid detection of genome, preparing reaction mixture, performing rapid PCR, electrophoresis cycles (containing ethidium bromide), and thus investigating result in the UV system in less than 90 min. Based on these results, the multiplex PCR can be used for rapid and simultaneous differentiation of three bacteria: *Salmonella typhi*, *Bacillus anthracis*, and *Yersinia pestis * from other similar bacteria. Given that these three factors can be named as biological and dangerous weapons, this study can provide a tool for rapid, accurate and low cost detection to detect them in case of bioterrorism operations and biological warfare. 
